# Antagonistic Antiviral Activity between IFN-Lambda and IFN-Alpha against Lethal Crimean-Congo Hemorrhagic Fever Virus *In Vitro*

**DOI:** 10.1371/journal.pone.0116816

**Published:** 2015-02-18

**Authors:** Licia Bordi, Eleonora Lalle, Claudia Caglioti, Damiano Travaglini, Daniele Lapa, Patrizia Marsella, Serena Quartu, Zoltan Kis, Kevin K. Arien, Hartwig P. Huemer, Silvia Meschi, Giuseppe Ippolito, Antonino Di Caro, Maria R. Capobianchi, Concetta Castilletti

**Affiliations:** 1 Laboratory of Virology, National Institute for Infectious Diseases “L. Spallanzani”, Rome, Italy; 2 Division of Virology, National Center for Epidemiology, Budapest, Hungary; 3 Unit of Virology, Institute of Tropical Medicine, Antwerp, Belgium; 4 Department of Hygiene, Microbiology & Social Medicine, Medical University Innsbruck, Innsbruck, Austria; 5 Scientific Direction, National Institute for Infectious Diseases “L. Spallanzani”, Rome, Italy; 6 Laboratory of Microbiology and Biorepository, National Institute for Infectious Diseases “L. Spallanzani”, Rome, Italy; Division of Clinical Research, UNITED STATES

## Abstract

**Background and Aims:**

Crimean Congo Hemorrhagic fever virus (CCHFV) is the causative agent of Crimean-Congo hemorrhagic fever, a severe disease with a mortality rate of around 30% in humans. Previous studies demonstrate that pre-treatment with type I IFNs have an antiviral effect against CCHFV, while established CCHFV infection is almost insensitive to subsequent IFN-α treatment. No data concerning type III IFNs antiviral activity against CCHFV are available so far. The aim of the present study was to explore the capability of IFN-λ1 to inhibit the replication of CCHFV and the possible synergism/antagonism between IFN-α and IFN-λ1 both in the inhibition of CCHFV replication and in the activation of intracellular pathways of IFN response.

**Methods:**

Human A549 and HuH7 cells were treated with increasing amounts of IFN-λ1, or IFN-α or a combination of them, infected with CCHF; the extent of virus yield inhibition and the induction of MxA and 2’-5’OAS mRNA was measured.

**Results and Conclusions:**

Our study pointed out that type III IFN possess an antiviral activity against CCHFV, even if lower than type I IFN. Moreover, a clear antagonism between IFN-λ and IFN–α was observed in both cell lines (A549 and HuH7 cells), in terms of antiviral effect and activation of pivotal ISGs, i.e. MxA and 2’-5’OAS. Elucidating the interplay between type I and III IFNs will help to better understand innate defence mechanisms against viral infections and may provide novel scientific evidence for a more rational planning of available and future treatments, particularly against human diseases caused by high concern viruses.

## Introduction

Crimean-Congo Hemorrhagic fever virus (CCHFV) is an emerging virus belonging to the Nairovirus genus of the *Bunyaviridae* family. It is the causative agent of Crimean-Congo hemorrhagic fever, a severe disease with a mortality rate of around 30% in humans, most of the deaths occurring 5 to 14 days after the onset of illness [1,2]. CCHFV is maintained in vertical and horizontal transmission cycles involving ixodid ticks and a variety of wild and domestic vertebrates, which do not show signs of illness. The virus circulates in a number of tick genera, but Hyalomma ticks are the principal source of human infection. Human-to-human transmission can occur, resulting from close contact with blood, secretions, or other body fluids from infected persons or by direct contact with virus-contaminated blood; nosocomial transmission and organ transplant-related transmission have been reported as well [3–6]. The virus shows a wide geographic distribution and is regarded as a public health threat in many regions of the word, including Asia, Eastern Europe, the Middle East, Africa and Russia [1,6,7]. Despite a wide distribution, the pathogenesis of CCHF remains poorly understood, because of limited human pathology and the need for high-containment facilities to handle CCHFV-infected specimens. There is currently no FDA-approved vaccine or specific antiviral therapy for CCHF. The classification of CCHFV as a WHO Risk Group IV pathogen and the lack of suitable animal models has caused progress in developing new prophylactic and therapeutic measures to be slow [8]. The innate immune response represents the first line of defence against viral infections in mammalian cells [9,10]. Upon sensing molecules produced during viral replication, signalling pathways are activated leading to induction and secretion of type I interferons (IFNs), mainly IFN-α and IFN-β, and subsequent upregulation of interferon stimulated genes (ISGs) [11]. Previous studies demonstrate that pre-treatment with type I IFNs have an antiviral effect against CCHFV [12], most likely due to the activities of IFN-induced antiviral proteins such as MxA [13,14], while established CCHFV infection is almost insensitive to subsequent IFN-α treatment [15]. Recently, a novel group of IFNs has been discovered (IFN-λ1/interleukin-29 [IL-29], IFN-λ2/IL-28A, and IFN-λ3/IL-28B) [16], and assigned to a new type (type III) of IFN. The biological activity of type III IFN overlaps to some extent with that of type I IFN, and similar subsets of ISGs are induced, although type III IFN exerts its action through a receptor complex distinct from that of type I IFN [17,18]. However, no data concerning type III IFNs antiviral activity against CCHFV are available so far.

Moreover, little is known about the effect of type I and type III IFNs combination, as available studies report contrasting information [19–21]. In a previous study from our group an antagonistic activity of IFN-λ1 and IFN-λ2 towards both the IFN-α-driven inhibition of replication of several viruses (EMCV, WNV lineage 1 and 2, HSV-1 and CHIKV) and the induction of ISG mRNA was described [22]. Furthermore, in a recently published study IFN-λ pre-treatment of human fibroblasts resulted in lower IFN-α signalling and pro-inflammatory ISGs induction in response to CMV Infection [23].

The aim of the present study was to explore the capability of IFN-λ1 to inhibit the replication of CCHFV in cell cultures and to investigate the possible synergism/antagonism between IFN-α and IFN-λ1 both in the inhibition of CCHFV replication and in the activation of intracellular pathways of IFN response.

## Materials and Methods

A549 (ICLC Cell Factory IST, Genova, Italy) and HuH7 (kindly provided by Marco Tripodi, Rome, Italy and purchased from the American Type Culture Collection) were maintained in Dulbecco’s Modified Eagle Medium (D-MEM), both containing 10% Foetal Calf Serum (FCS) and penicillin/streptomycin at 37°C in a humidified atmosphere of 5% CO_2_.

For virus stock preparation, A549 and HuH7 cells were infected with CCHFV strain 10200 (kindly provided by Anna Papa, Thessaloniki, Greece). Cell lysates were clarified, aliquoted, and stored at −80°C until use. Virus titration was performed on the respective host cell line by limiting dilution assay, by determining tissue culture infectious dose (TCID_50)_ with the method of Reed & Muench. [24]. Virus stock titers were: 10^8.25^TCID_50_/ml for CCHFV on A549 and 10^6.75^TCID_50_/ml CCHF on HuH7. All procedures involving infectious CCHFV were performed in a biosafety level 4 (BSL4) facility, according to standard operating procedures approved by the institutional biosafety committee. This study was carried out as part of an international training programme, involving Biosafety Level 4 Laboratories from different countries, aimed at sharing and improving Group 4 viruses safety management.

The following IFN preparations were used: human recombinant IFN-α2b (Intron; Schering Corp., Kenilworth, NJ, USA; specific activity: 400MIU/mg,1IU corresponding to 2.5pg), human recombinant IL-29/IFN-λ1 (R&D Systems; Inc., Minneapolis, USA; ED_50_ 1–5ng/mL, 1IU corresponding to 1–5 ng).

A549 and HuH7 cells were seeded at about 4/5x10^4^ cells per well in 96-well plates and after 1 day were incubated with increasing amounts of either recombinant IFN-α (1, 10, 10^2^ and 10^3^IU/mL) or IFN-λ1 (0.01, 0.1, 1 and 10ng/mL) for 18–20h. In parallel cultures, the cells were incubated with mixtures of IFN-α+IFN-λ1 (1IU/mL+0.01ng/mL, 10IU/mL+0.1ng/mL, 10^2^IU/mL+1ng/mL and 10^3^IU/mL+10ng/mL, respectively). Cell monolayers were then infected with CCHFV at a multiplicity of infection (MOI) of 0.01 TCID_50_/cell and after 24h culture supernatants were collected and titrated to assess virus yield. These experimental conditions (low MOI and overnight incubation, implying multiple replication cycles) were selected in order to maximize the inhibitory effect of IFNs, and to obtain a higher resolution in the virus yield inhibition curves.

Combination index (CI) for constant ratio combinations was calculated through the CompuSyn software, which uses the CI isobologram method of Chou and Talalay [25]. The interpretation of CI value in quantifying two drug antiviral interactions is as follows: CI = 1, additive; CI>1, antagonism; CI<1, synergism.

The quantification of MxA and 2’-5’OAS mRNA was performed on total cellular RNA, extracted using Trizol (Gibco BRL, Grand Island, NY, USA), by a Taqman real-time RT-PCR method previously established in our laboratory. In particular, for each gene (including β-actin as housekeeping gene) a standard curve was prepared with serial dilutions of a recombinant plasmid containing the region of interest [26]. For each experimental condition, the ratio of mRNA for MxA and 2’-5’OAS to β-actin was calculated, and the results were expressed as fold induction over untreated cells

## Results and Discussion

**Fig 1:**
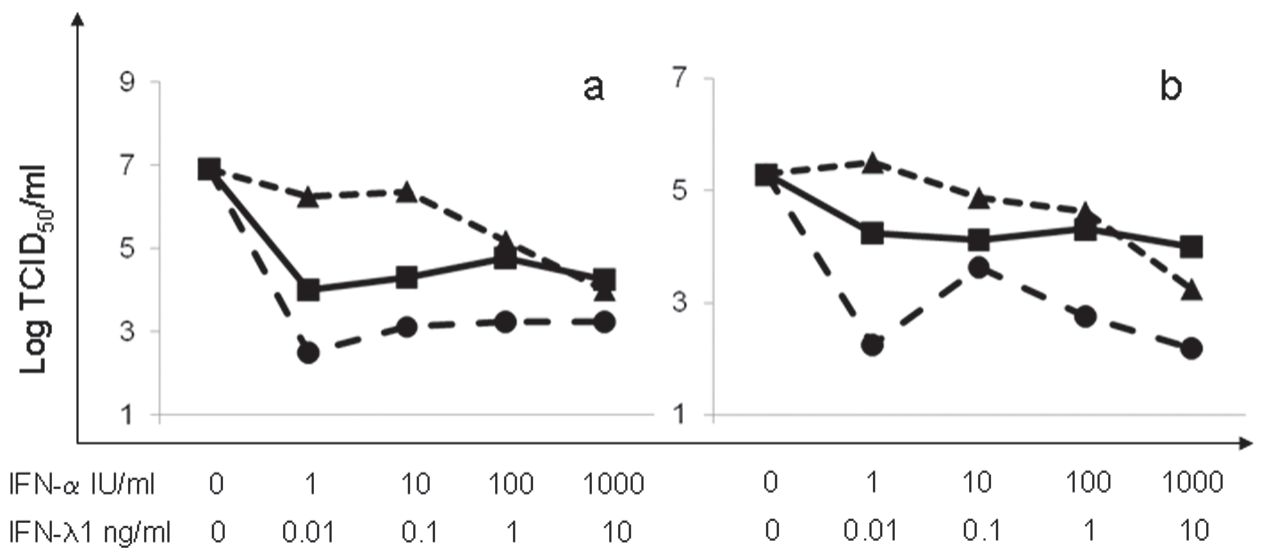
Dose-dependent inhibition of CCHFV replication by recombinant IFN-α, IFN-λ1 and IFN-α+IFN-λ1. A549 **(a)** and HuH7 cells **(b)** were treated for 1 day with increasing amounts of either IFN type, alone or in combination, then infected with CCHFV at MOI 0.01; infectious virus yield was measured after overnight incubation. One out of three experiments is shown. Dotted lines: IFN-α (●) or IFN-λ1 (▲) used alone; continuous line: IFN-α and IFN-λ1 (■) used in combination.

The ability of human recombinant IFN-α and IFN-λ1 to inhibit the replication of CCHFV in different cell lines was initially investigated. Although virus yield varied between the 3 experiments performed, the overall trend of the results was uniform, showing a substantially different ability of the two IFN types to inhibit CCHFV replication, both in A549 (Fig. 1a) and HuH7 cells (Fig. 1b). In Fig. 1a and b, one representative experiment is reported, showing that both IFN-α and IFN-λ1, used alone, reduced CCHFV yield in a dose-dependent manner, being IFN-α more potent than IFN-λ1.

When considering the antiviral effects of combinations of IFN-α with IFN-λ1, for each combination the inhibition of CCHF yield was invariably smaller than that obtained for IFN-α alone (Fig. 1a and b) indicating antagonistic effect between the two IFN types; notably, for the highest dose (10^3^IU/mL IFN-α+10ng/mL IFN-λ1) the inhibition was even smaller than that obtained with IFN-λ1 alone in both A549 and HuH7 cells (Fig. 1a and b).

The antagonistic effect was statistically confirmed by calculating the Combination index for each combination. As shown in Table 1, CI was well above 1 for all IFN-α + IFN-λ1 combinations, both in A549 and HuH7 cells, adding strong statistical support to the graphical results shown in Fig. 1.

**Table 1:** Combination index for IFN-α and IFN-λ1 against CCHF replication respectively in A549 and HuH-7 cells.

IFN-α	IFN-λ1	Combination Index*	
(IU/ml)	(ng/ml)	A549	HuH-7
1.0	0.01	582.8	1.08E14
10.0	0.1	103,469	3.04E13
100.0	1.0	9.25E7	3.55E20
1000.0	10.0	5,008,013	1.19E14

*The Combination Index (CI) was calculated using the CompuSyn software (Chou, T.-C. and Martin, N. CompuSyn software for drug combinations and for general dose effect analysis, and user’s guide. ComboSyn, Inc. Paramus, NJ 2007. [www.combosyn.com]) which uses the method of Chou & Talalay. CI values <1, 1 and >1 indicate synergism, additive effect and antagonism, respectively.

In order to characterize possible mechanisms underlying the observed antagonism between IFN-α and IFN-λ1, we addressed the expression of two well-know IFN-inducible proteins, MxA and 2’-5’OAS. To this aim, HuH7 and A549 cells were incubated for 3, 6 an 24h, a with the same IFN doses (either alone or in combination) used for the virus inhibition experiments. At the indicated time points the quantification of MxA and 2’-5’OAS mRNA was performed.

**Fig 2:**
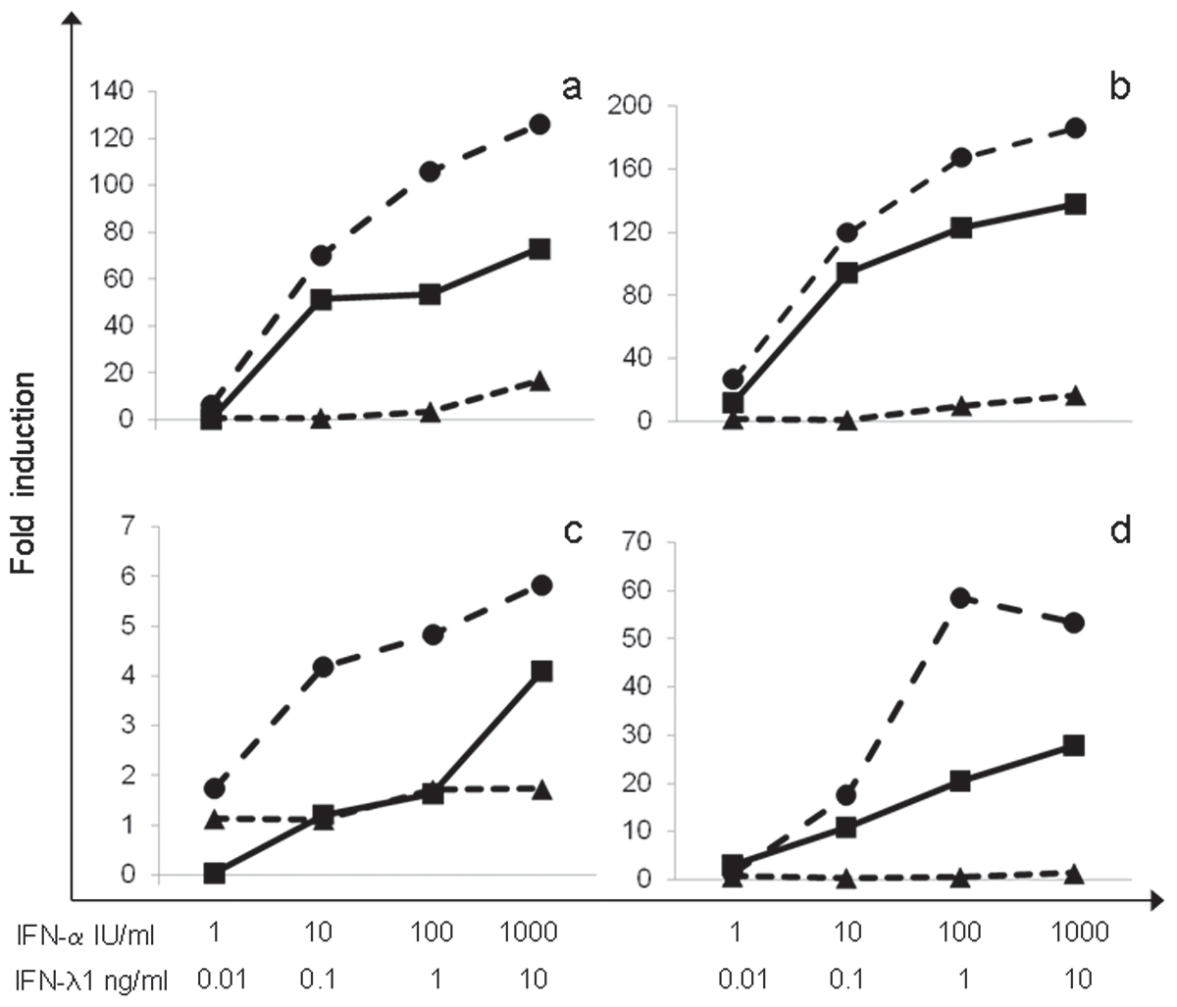
Dose-dependent induction of MxA and 2’-5’OAS mRNA levels following treatment with recombinant IFN-α, IFN-λ1 and IFN-α +IFN-λ1. A549 **(a), (c)** and HuH7 cells **(b), (d)** were exposed 3h to increasing amounts of either IFN type, alone or in combination, then mRNA levels for MxA **(a), (b)** and 2’-5’OAS **(c), (d)** were measured, expressed as fold induction vs. untreated cells. One out of three experiments is shown. Dotted lines: IFN-α (●) or IFN-λ1 (▲) used alone; continuous line: IFN-α and IFN-λ1 (■) used in combination.

A dose- and time-dependent induction of mRNA for both ISGs was observed in both cell lines treated with the single IFN types, being again IFN-α much more effective than IFN-λ1. One representative (out of 3) experiment is shown in Fig. 2. The results were in close agreement with those obtained in virus yield inhibition experiments, indicating a weaker effect of IFN-λ1 as compared to IFN-α; once again, all the IFN combinations were less effective than IFN-α alone, supporting antagonism between the two IFN types.

In conclusion our study pointed out, for the first time, that type III IFN (IFN-λ1) possess an antiviral activity against CCHFV, even if lower than type I IFN (IFN-α2b). Moreover, a clear antagonism between IFN-λ and IFN–α was observed in two different cell lines (A549 and HuH7 cells), in terms of both antiviral effect and activation of pivotal ISGs, i.e. MxA and 2’-5’OAS.

Although possible mechanisms underlying the antagonistic effects of IFN-λ remain to be elucidated, it is possible that differences in the kinetics of activation of intracellular pathways involved in antiviral activity may play an important role. In fact, the kinetics of induction of ISGs differs between the 2 cytokines, as IFN-λ acts slowly but leads to a steady increase in ISGs induction, whereas IFN-α not only induces ISGs rapidly, but also leads to their rapid decline [19,27,28]. In turn, the more rapid, but less intense, STAT activation and ISGs induction by IFN-λ may lead to a partial desensitization to IFN-α, consistent with a recent study where UPS18 was found to mediate the inhibition of IFN-α activities by IFN-λ [29].

Elucidating the interplay between type I and III IFNs will help to better understand innate defence mechanisms against viral infections and may provide novel scientific evidence for a more rationale planning of available and future treatments, particularly against human diseases caused by high concern viruses.
